# Effect of interaural electrode insertion depth difference and independent band selection on sentence recognition in noise and spatial release from masking in simulated bilateral cochlear implant listening

**DOI:** 10.1007/s00405-023-07845-w

**Published:** 2023-01-25

**Authors:** Hasna Fathima, Jayashree S. Bhat, Arivudai Nambi Pitchaimuthu

**Affiliations:** 1 Department of Audiology and Speech-Language Pathology, Kasturba Medical College, Mangalore, Manipal Academy of Higher Education, Manipal, Karnataka 575001 India; 2 Department of Audiology and Speech-Language Pathology, National Institute of Speech and Hearing, Trivandrum, Kerala 695017 India; 3 Kasturba Medical College Hospital, Ambedkar Circle, Mangalore, Karnataka 575001 India

**Keywords:** Spatial release from masking, Bilateral cochlear implant, Binaural hearing, Vocoder processing, Advanced combined encoder, ‘n-of-m’ processing

## Abstract

**Purpose:**

Inter-aural insertion depth difference (IEDD) in bilateral cochlear implant (BiCI) with continuous interleaved sampling (CIS) processing is known to reduce the recognition of speech in noise and spatial release from masking (SRM). However, the independent channel selection in the ‘n-of-m’ sound coding strategy might have a different effect on speech recognition and SRM when compared to the effects of IEDD in CIS-based findings. This study aimed to investigate the effect of bilateral ‘n-of-m’ processing strategy and interaural electrode insertion depth difference on speech recognition in noise and SRM under conditions that simulated bilateral cochlear implant listening.

**Methods:**

Five young adults with normal hearing sensitivity participated in the study. The target sentences were spatially filtered to originate from 0° and the masker was spatially filtered at 0°, 15°, 37.5°, and 90° using the Oldenburg head-related transfer function database for behind the ear microphone. A 22-channel sine wave vocoder processing based on ‘n-of-m’ processing was applied to the spatialized target-masker mixture, in each ear. The perceptual experiment involved a test of speech recognition in noise under one co-located condition (target and masker at 0°) and three spatially separated conditions (target at 0°, masker at 15°, 37.5°, or 90° to the right ear).

**Results:**

The results were analyzed using a three-way repeated measure analysis of variance (ANOVA). The effect of interaural insertion depth difference (*F* (2,8) = 3.145, *p* = 0.098, *ɳ*^2^ = 0.007) and spatial separation between target and masker (*F* (3,12) = 1.239, *p* = 0.339, *ɳ*^2^ = 0.004) on speech recognition in noise was not significant.

**Conclusions:**

Speech recognition in noise and SRM were not affected by IEDD ≤ 3 mm. Bilateral ‘n-of-m’ processing resulted in reduced speech recognition in noise and SRM.

## Introduction

Everyday listening often involves selective attention to a specific speaker in the presence of multiple competing speech signals. The spatial separation between the sound sources is one of the cues for perceptual segregation of the target from masker(s) [2–4]. Interaural time difference (ITD) and interaural level difference (ILD) of sounds are the binaural cues for segregating the target from the masker based on their localization in the horizontal plane [5]. The improvement in speech recognition performance when a co-located noise is separated from the target is called spatial release from masking (SRM) [6–9].

Currently, the bilateral cochlear implant (BiCI) is the best treatment option for bilateral severe to profound hearing loss [10]. Bilateral implantation improves speech recognition in quiet [11]. However, the binaural advantages of bilateral implantation such as speech recognition in noise and horizontal localization are poor when compared to normal hearing listeners [12–14]. Binaural coherence or the inter-aural similarity in spectro-temporal characteristics of the input is a prerequisite for the accurate encoding of ITD and ILD cues in the auditory system [15, 16]. Binaural coherence in BiCI is compromised by various factors such as the nature of sound encoding strategy [17], mismatches in electrode position, [18, 19], and/or, electrode deactivation [20, 21].

The auditory system is tonotopically organized from the cochlea to the auditory cortex, and the binaural neuronal system computes the interaural time difference (ITD) and interaural level difference (ILD) from the tonotopically matched frequency bands of binaural inputs [22]. Multi-channel cochlear implants attempt to mimic the cochlea's tonotopicity by presenting electrical stimulation through electrodes placed at different sites in the cochlea. The electrode array’s insertion depth determines the place of stimulation. Data from in vivo computerized tomography (CT) scans in CI users revealed the array insertion depths range of 11.9–25.9 mm and the estimated frequency stimulated by the most apical electrode ranged from 308 to 3674 Hz [23]. The depth of insertion of the electrode is associated with frequency-place mismatch and reduction in speech recognition score. Deeper insertions resulted in more detrimental effects compared to shallower insertions [21]. The inter-aural electrode insertion depth is not precisely matched during bilateral cochlear implant (BiCI) surgery and incidences of inter-aural electrode insertion depth differences (IEDD) are not uncommon [23–28]. The inter-aural mismatch in the frequency allocation resulting from IEDD might result in inadvertent inter-aural frequency mismatches in the neural inputs to the binaural system [29]. A review of CT scans of 107 BiCI users showed a median IEDD of 1.3 mm and greater than 3 mm IEDD in 13–19% of the subjects [30]. The IEDD derived from CT reports of BiCI users has been correlated with perceptual measures of inter-aural time difference threshold [28]. Perceptual consequences of IEDD on binaural processing have been studied using speech and non-speech stimuli. An IEDD of more than 3 mm has been found to interfere with binaural fusion, horizontal lateralization [19, 29], speech recognition in noise, and SRM [18, 31].

Another important factor that can result in binaurally incoherent inputs is the sound coding strategy used in BiCI. Some strategies select the ‘n’ number of bands with the highest amplitude out of a total of ‘m’ bands, also known as ‘n-of-m’ processing [32]. When implemented in BiCI, the ‘n-of-m’ processing of the electrodes stimulated in right CI might be different from that of the left CI. This could result in interaural spectral differences and thereby reduce the binaural coherence. The effect of bilateral ‘n-of-m’ processing on the encoding of binaural cues is sparsely studied. Using an objective method of analysis on a manikin that wore a BiCI, Kan et al. [17] reported that ‘n-of-m’ processing resulted in inaccurate ITD encoding. In BiCI users, independent band selection has been found to reduce the sentence recognition score (SRS) in noise compared to binaurally linked band selection [33]. However, the effect of independent band selection on the SRM has not been studied. Also, the previous studies on the effects of IEDD on speech recognition and SRM were carried out only for sound coding strategies based on continuous Interleaved Sampling (CIS). Unlike the ‘n-of-m’ strategy which results in independent band selection, the CIS- strategy processes all the bands and therefore by itself does not add inter-aural spectral differences. Thus, the effect of IEDD in the ‘n-of-m’ coding strategy could be presumably different from that on CIS.

The present study aimed to investigate the effect of bilateral ‘n-of-m’ processing strategy and interaural electrode insertion depth difference on speech recognition in noise and SRM under conditions that simulated bilateral cochlear implant listening. We hypothesized that the independent band selection in bilateral ‘n-of-m’ processing would reduce the SRM and the presence of IEDD would result in further reduction in the performance. Studies on actual CI users are affected by inter-subject variabilities in the degree of neuronal survival, language development, device settings, etc. [34]. Vocoder simulations allow flexible investigation of factors that cannot be otherwise altered in CI users such as the changes in IEDD. Therefore, sine wave vocoders were implemented to simulate some of the characteristics of cochlear implant signal processing in this study. Speech recognition in noise and SRM were chosen as the experimental tools in this study because of the reported utility of these tools to study the effect of interaural frequency mismatches on binaural processing in BiCI [17, 28, 32, 33, 35, 36].

## Methods

### Participants

Five normal-hearing young adults who are native speakers of the Kannada language were recruited for the study by purposive sampling. According to the model proposed by Anderson and Vingrys [37] for psychophysical research, if the participants are recruited from a selectively normal population and a non-equivocal effect is observed in all the participants, a sample size of five is enough for ascertaining whether the effect is present in more than 50% of the population. The present study fulfills the assumptions of the model and therefore the sample size used in the study meets the minimum required number of participants. The age of the participants ranged from 20 to 23 years (mean age: 21.4 years, standard deviation: 1.34). The hearing thresholds of the participants were ≤ 25 dBHL at octave audiometric test frequencies from 250 Hz to 8 kHz. None of the participants had any history of middle ear disorders. The institutional ethics committee has approved the study (approval number: 09/2020/250). All the experiments were conducted as per the Declaration of Helsinki [38]. Informed consent was obtained from all the participants before conducting the study.

### Stimuli and equipment

The target sentences for the experiment were taken from a standardized Kannada sentence list [39]. There were 25 lists and each list had ten sentences. The practice items were taken from the Quick-SIN Kannada sentence list [40]. A 4-talker babble recorded in the Kannada language served as the masker. The stimuli were recorded in Praat software [41] uttered by a female speaker. Vocoder processing and spatial filtering were applied using the MATLAB R2020b platform. The processed stimuli were presented to the participants from a laptop (MacBook Pro) using Sennheiser HD280-pro circum-aural headphones (Sennheiser, Wedemark, Germany) routed via Motu 16A audio interface. The sampling frequency of the target and masker was 44,000 Hz.

### Signal processing

#### Spatial filtering

The non-individualized head-related transfer function (HRTF) corresponding to the ‘BTE-front” microphone measurement from the Oldenburg HRTF database [42] was applied separately to the target and the masker. The target was filtered at 0° azimuth and the masker at 0°, 15°, 37.5°, and 90° azimuth. The spatialized target and masker were added together to generate four conditions which are as follows: (a) both target and masker filtered at 0° azimuth (b) the target at 0° and masker at 15° (c) the target at 0° and the masker at 37.5° and (c) the target at 0° and the masker at 90°.

#### Vocoder processing

The purpose of vocoding was to simulate cochlear implant listening. For this research, a vocoder corresponding to 22 channel cochlear implant was generated using MATLAB-2020 (MathWorks. Inc. Natick, MA). The signals were pre-emphasized above 2000 Hz by passing through a first-order Butterworth filter. The signals were bandpass filtered into twenty-two channels in the forward and backward direction using a window-based finite impulse response (FIR) filter of the order 1024. The edge frequencies and center frequencies of each passband were derived using the Greenwood function [43]. The envelope of the signal was extracted by half-wave rectification and low pass filtering with a second-order Butterworth filter with a 400 Hz cut-off frequency. The envelope of each band was multiplied by a sine wave corresponding to the center frequency of the synthesis band based on the Greenwood function [43]. The Root Mean Square (RMS) amplitude of each band was calculated and arranged in descending order. Based on the RMS value of the bands, eight bands with the highest RMS amplitude values are selected. The filtered waveforms of the selected bands are summed. The vocoder simulation of cochlear implant listening with the ‘n-of-m’ strategy is usually performed on time frames of 8 ms length on a windowed signal [42, 44, 45]. The band selection is performed on each frame and finally, the frames are padded together. The objective of the present study was to investigate the effect of independent band selection on binaural coherence. Therefore, band selection was performed on the entire sentence. A band selection on a windowed signal with a frame size of 4–8 ms ideally simulates the real-time processing in cochlear implants, however, in that case, direct inter-aural comparisons on the differences in channel selection resulting from the RMS criteria would be difficult to obtain.

##### Simulation of inter-aural electrode insertion depth difference (IEDD)

The interaural mismatches in place of stimulation related to insertion depth differences of 1.5 mm and 3 mm towards the base were simulated by corresponding upward shifts in the center frequency of the synthesis bands. For the 0-IEDD condition, the center frequency allocation in the right and left vocoder were equal to the analysis band’s center frequency. For simulating IEDD, the center frequencies of the right vocoder were unchanged, and that of the left vocoder was shifted upward by a value equal to ∆f. The center frequencies of the synthesis bands in the left vocoder corresponding to the insertion depth difference conditions of 0 mm, 1.5 mm, and 3 mm are given in Table 1.

**Table 1:** The band-specific carrier frequencies corresponding to the IEDD of 0 mm, 1.5 mm, and 3 mm for the left vocoder used in the study

Band	0 mm shift	1.5 mm shift	3 mm shift
1	99.63648909	156.1620772	225.7307695
2	142.3285609	208.6930539	290.3682036
3	192.4541505	270.3701186	366.2585108
4	251.3075775	342.7856827	455.3607285
5	320.4085278	427.80941	559.9749473
6	401.5412938	527.6364884	682.8016855
7	496.8008481	644.8443069	827.0116009
8	608.6469385	782.4589994	996.3273376
9	739.9676028	944.0335756	1195.119623
10	894.1537418	1133.739654	1428.520095
11	1075.186678	1356.475168	1702.553769
12	1287.740961	1617.990822	2024.294568
13	1537.305066	1925.038566	2402.04793
14	1830.323124	2285.545924	2845.565206
15	2174.361308	2708.820667	3366.295381
16	2578.303214	3205.791127	3977.680616
17	3052.579241	3789.288355	4695.503253
18	3609.435923	4474.377393	5538.293211
19	4263.252155	5278.746236	6527.806327
20	5030.910476	6223.162509	7689.585959
21	5932.232999	7332.009654	9053.622361
22	6990.493257	8633.916481	10,655.12686

### Procedure

#### Practice trials

The participants underwent practice trials for avoiding the learning effects which are usually reported with spectrally shifted vocoded sentences [46]. The practice task involved the recognition of sentences in the presence of a four-talker babble. Forty-nine sentences were presented from the Quick-SIN Kannada sentence list [40]. The number of keywords in each sentence was five. For each list, the first sentence was presented at an SNR of 20 dB SPL and for each sentence that followed, the SNR was reduced by 5. Therefore, the last item in any list had − 15 dB SNR. The SNR at which 50% of the keywords were correctly recognized was estimated as SNR50. Obtaining a plateau response for SNR50 during the practice was considered as the evidence for saturation of the practice effects.

#### Experiment

The perceptual experiment consisted of sentence recognition in noise under three SNR conditions (-10 dB SNR, 0 dB SNR and + 10 dB SNR), across four spatial conditions (one co-located and three spatially separated) and three IEDD conditions (0, 1.5, and 3 mm). The order of experimental conditions was randomized. The first 25 conditions were tested using randomly selected lists from a standardized Kannada sentence list [39]. The remaining eleven conditions were randomly chosen from the list of 25 and consideration was given to those lists which yielded poorer scores. The participants wearing circum-aural headphones were seated comfortably in front of a laptop in a sound-treated room. They were instructed to repeat what is heard through the headphone. Guessing was permitted. The verbal responses were recorded using Praat [41] for further analysis. The approximate length of practice was 30 min, and the experiment was 90 min. A 5-min break was provided every 30 min.

#### Scoring

Each correctly identified keyword was assigned a score of one. The maximum possible raw score per list was forty. The SRS was obtained by converting the raw score into a percentage by multiplying by 2.5. For example, a raw score of 40 would result in an SRS of 100%. For statistical analysis, the SRS was converted to rationalized arcsine units (RAU score) by using the following equation:

1 $$t=2\times \mathrm{asin}(\sqrt{p})$$

$$\mathrm{RAU score}=\left(46.4732\times t\right)-23$$

where *p* is the percentage of correct responses converted into a value between 0 and 1.

The SRM was calculated as the difference between the RAU scores obtained for co-located conditions and spatially separated conditions.

#### Statistical analysis

Statistical analysis was performed using SPSS25.0 (SPSS Inc, Chicago, USA) Three-way repeated measure analysis of variance (ANOVA) was performed to analyze the main effect of the three independent variables which were the IEDD, the azimuthal separation between target and masker (*A*_mt)_, and the SNR on SRS and SRM. Post hoc comparisons were done using two-tailed, paired *t* tests.

## Results

### Effect of interaural electrode insertion depth difference on SRS

**Fig. 1:**
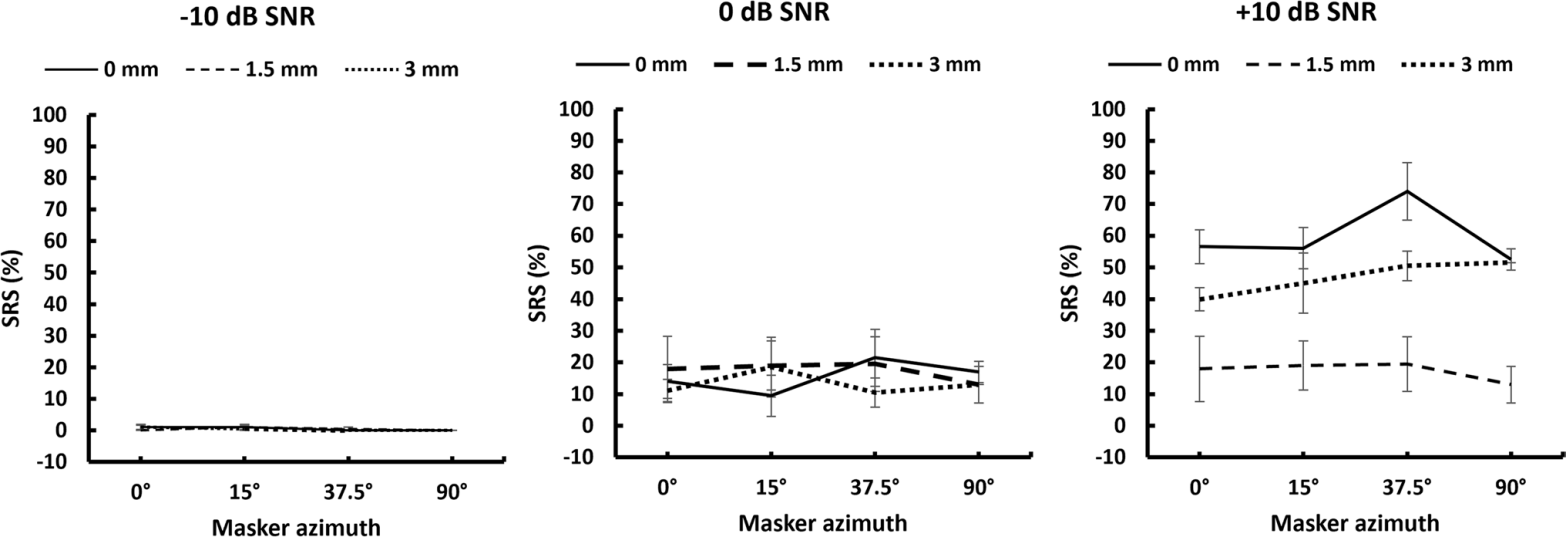
The mean and standard error of the mean of SRS (± 1 standard error) obtained for the IEDD of 0 mm, 1.5 mm, and 3 mm for the − 10, 0, and + 10- SNR conditions are plotted for the co-located condition having target and masker at 0°, and spatially separated conditions with the target at 0° and masker at 15°, 37.5° or 90° to the right ear

Three-way repeated measure ANOVA did not reveal statistically significant main effect of IEDD (*F* (2,8) = 3.145, *p* = 0.098, *ɳ*^2^ = 0.007), and the *A*_mt_ (*F* (3,12) = 1.239, *p* = 0.339, *ɳ*^2^ = 0.004) on SRS. However, the effect of SNR on SRS was statistically significant (*F* (2,8) = 64.499, *p* =  < 0.001, *ɳ*^2^ = 0.796). There was no significant interaction between the IEDD and *A*_mt_ (*F* (6,24) = 0.756, *p* = 0.611, *ɳ*^2^ = 0.004), IEDD and SNR (*F* (4,16) = 2.847, *p* = 0.059, *ɳ*^2^ = 0.007), *A*_mt_ and SNR (*F* (6,24) = 1.415, *p* = 0.250, *ɳ*^2^ = 0.006), and IEDD, *A*_mt_ and SNR (*F* (12,48) = 0.793, *p* = 0.656, *ɳ*^2^ = 0.010). Figure 1 represents the mean and standard error of the mean of SRS obtained for − 10, 0, and + 10 SNR for the IEDD of 0 mm, 1.5 mm, and 3 mm.

**Fig. 2:**
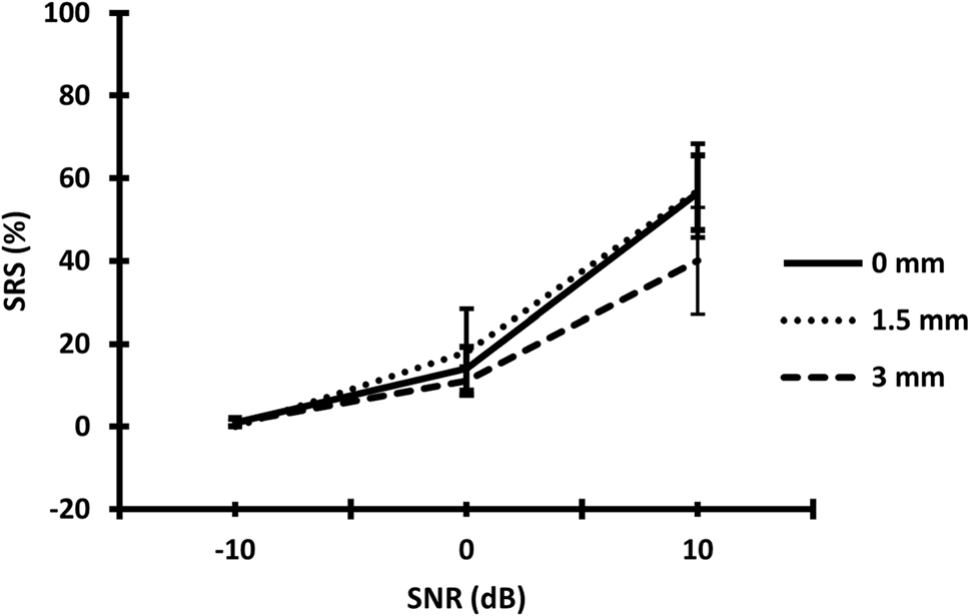
The mean and standard error of the mean of SRS (± 1 standard error) obtained for − 10, 0, and + 10-SNR conditions for the IEDD of 0 mm, 1.5 mm, and 3 mm for the co-located condition

Since the IEDD and *A*_mt_ were found to have no statistically significant effect on SRS, post hoc comparisons between the SNR conditions with Bonferroni’s correction were done on pooled data. The mean SRS improved with increase in SNR from -10 SNR to 0 SNR (*t* = − 4.485, *p* =  < 0.006), from − 10 SNR to 10 SNR (*t* = − 11.279, *p* =  < 0.001), and from 0 to 10 SNR (*t* = − 6.794, *p* =  < 0.001). Figure 2 represents the mean and standard deviation of SRS obtained for the co-located condition under the three SNR conditions used in the study.

### Effect of interaural electrode insertion depth difference on SRM

**Fig. 3:**
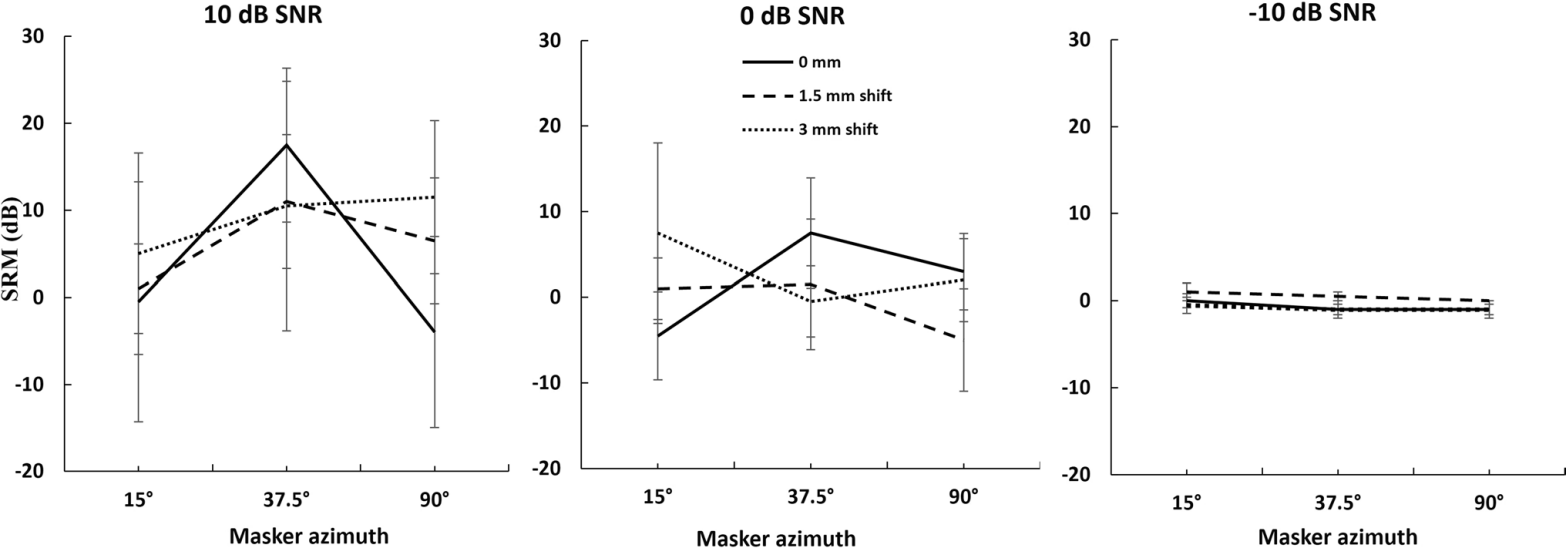
Mean and standard error of the mean of SRM(dB) obtained for azimuthal separation of 15°, 37.5°, and 90° from the target at 0° for the IEDD of 0 mm, 1.5 mm, and 3 mm for the − 10, 0, and + 10- SNR conditions

Three-way repeated measure ANOVA did not reveal statistically significant main effect of IEDD (*F* (2,8) = 0.099, *p* = 0.907, *ɳ*^2^ = 0.003), *A*_mt_ (*F* (2,8) = 2.017, *p* = 0.195, *ɳ*^2^ = 0.011) and the SNR (*F* (2,8) = 2.575, *p* =  < 0.137, *ɳ*^2^ = 0.045) on SRM. There were no significant interactions between the IEDD and *A*_mt_ (*F* (4,16) = 1.019, *p* = 0.427, *ɳ*^2^ = 0.015), IEDD and SNR (*F* (4,16) = 0.333, *p* = 0.852, *ɳ*^2^ = 0.028), *A*_mt_ and SNR (*F* (4,16) = 1.187, *p* = 0.354, *ɳ*^2^ = 0.021), and IEDD, *A*_mt_ and SNR (*F* (8,32) = 1.081, *p* = 0.401, *ɳ*^2^ = 0.042). The mean and standard error of the mean of SRM obtained for IEDD of 0 mm, 1.5 mm, and 3 mm are plotted for − 10, 0-, and + 10-dB SNR conditions in Fig. 3.

### Effect of ‘n-of-m’ processing on SRS and SRM

**Fig. 4:**
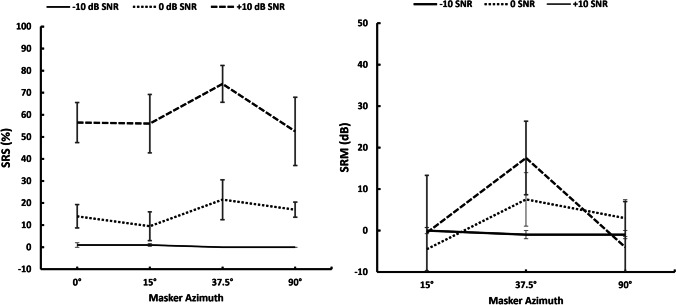
Mean and standard error of the mean of SRS and SRM obtained for − 10, 0, and + 10 SNR for the ‘n-of-m’ processing without IEDD

Two-way repeated measure ANOVA did not reveal a statistically significant main effect of the *A*_mt_ (*F* (3,12) = 1.920, *p* = 0.180, *ɳ*^2^ = 0.014) on SRS. However, the effect of SNR on SRS was statistically significant (*F* (2,8) = 62.505, *p* =  < 0.001, *ɳ*^2^ = 0.797). Also, the two-way repeated measure ANOVA did not reveal statistically significant main effect of the *A*_mt_ (*F* (2,8) = 2.384, *p* = 0.154, *ɳ*^2^ = 0.066) and SNR (*F* (2,8) = 0.476, *p* = 0.638, *ɳ*^2^ = 0.030) on SRM. Figure 4 represents the mean and standard error of the mean of SRS and SRM obtained for − 10, 0, and + 10 SNR across the azimuthal conditions.

## Discussion

This study investigated the effect of simulated IEDD, *A*_mt_, and SNR on speech recognition in noise and SRM. The speech recognition in noise was compared between the frequency-unshifted condition with simulated IEDD of 0 mm and that of frequency-shifted conditions with IEDD of 1.5 mm and 3 mm under co-located and three spatially separated conditions. The co-located condition had a horizontal azimuth of target and masker at 0° and spatially separated conditions had the target at 0° and masker being shifted from 0° to 15°, 37.5°, and 90°. The SNR tested were − 10, 0, and + 10.

The IEDD and *A*_mt_ did not affect speech recognition in noise and SRM in the present study. The SRS improved when the SNR was increased. However, the SRM was unaffected by SNR also. The findings are consistent with the previous study using simulated CI listening having an eight-band-vocoder implementation of continuous interleaved sampling (CIS) processing by Goupell et al. [18] which reported that IEDD of 3 mm or lesser does not affect speech recognition in noise and SRM. Also, in our study, the increase in target-masker spatial separation did not improve the scores even when the IEDD was absent, reflecting the loss of access to spatial segregation cues in bilaterally symmetrical electrode insertion depth conditions.

The IEDD results in an inter-aural mismatch in the carrier frequencies used for modulating the envelope and reduces the inter-aural envelope coherence which is a pre-requisite to the binaural processing [15, 47, 48]. IEDD has been reported to affect horizontal localization, speech recognition in noise, and SRM[15, 17, 18, 47, 49]. In addition to this, the monaural place-frequency mismatch also results in reduced speech intelligibility in both ears [20, 29, 48, 50, 51] and the effect on speech recognition will be more affected in the ear with deeper insertion [49, 50]. In this study, IEDD was introduced by an upward shifting of the carrier frequencies in the left ear. However, the effect of IEDD on speech recognition in noise and SRM was not observed for the conditions tested in this study. The findings are consistent with previous research on IEDD whereas the inter-aural frequency mismatches introduced by simulated electrode insertion depth differences were not affecting speech recognition in noise and SRM up to 3 mm of IEDD [15, 17, 18]. In addition, it is also possible that the listener must have relied on the speech information in the ear with a better signal [52] for performing the speech recognition task while ignoring the degraded information in the opposite ear, as observed previously for spectrally shifted [53] and unshifted vocoded stimuli [54].

### Effect of independent band selection in simulated bilateral CI listening on speech recognition in noise

In the present study, the vocoder processing involved simulation of the ‘n-of-m’ strategy. There were 22 analysis bands, out of which eight bands with the highest RMS amplitude were selected as synthesis bands. Only these selected bands were considered for further processing. In this study, the bilateral frequency-unshifted condition with IEDD of 0 mm was used to investigate the independent effect of the ‘n-of-m’ strategy. The findings of the study show that ‘n-of-m’ processing alone leads to diminished spatial cues for segregation.

**Fig. 5:**
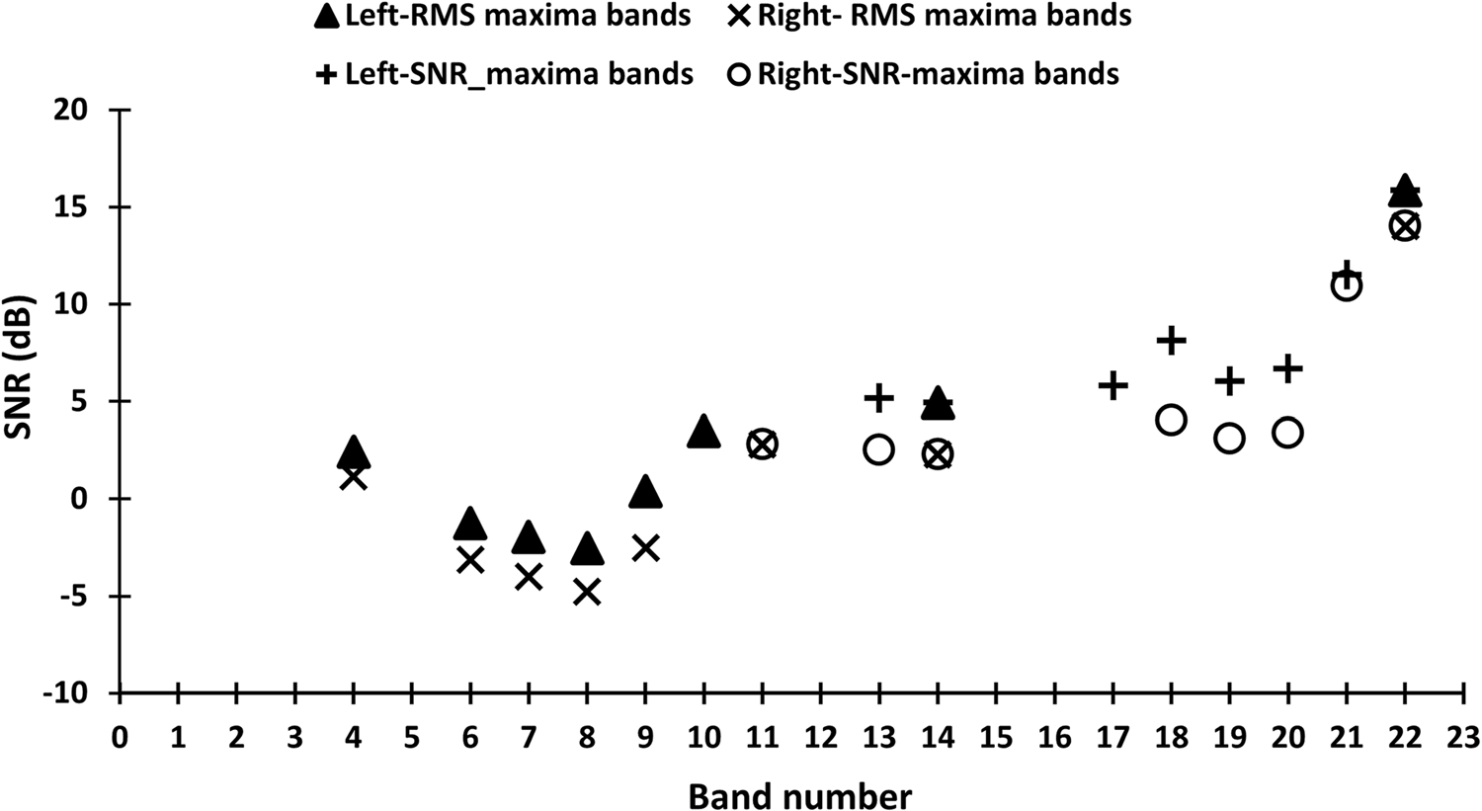
The SNR (dB) in the bands selected based on the RMS and the SNR. The IEDD was 0 mm and the location of the target was at 0° and that of the masker was at 90°

The ‘n-of-m’ processing based on RMS amplitude reduces the spatial cues in at least two different ways. Firstly, the band selection is ear-independent and binaurally unlinked. Therefore, the bands selected could differ across the right and left ears, especially when the target and masker are spatially separated. Interaural differences in the band selection can create interaural frequency differences. The second factor that can affect the performance in bilateral ‘n-of-m’ processing is the RMS-based criteria used for band selection. The RMS amplitude of the band is determined by the energy of the signal and masker. So it is not differentiated whether the energy in the band is dominated by signal or masker. If the band energy is dominated by noise rather than the signal, it can lead to the selection of frequency channels that has a low signal-to-noise ratio (SNR). The selection of bands having high RMS amplitude and low SNR can degrade the acoustic cues for speech recognition in noise. To demonstrate the lack of correlation between the RMS and SNR in the selected bands, the ratio of RMS of signal and RMS of noise was calculated for each band to derive SNR (dB) and plotted in Fig. 5. In each band, the SNR is plotted for the selected bands for the right and left vocoder for sentence number 16 from list 1 of the sentence list used in the present study. The target was filtered at 0° and the masker at 90° to the right at an SNR of zero. The IEDD was selected as zero to avoid the influence of IEDD. It can be noted that the SNR corresponding to the bands selected based on RMS amplitude is below zero for bands 6,7, and 8. There are inter-aural differences in the band selection for band numbers 10 and 11. The bands selected based on SNR criteria do not overlap with that selected based on RMS amplitude criteria except for the 11, 13, and14th bands. As evident in Fig. 5, better RMS amplitude does not translate to better SNR in the band.

The present study also points to the differences in the pattern of inter-aural frequency differences generated by IEDD and ‘n-of-m’ processing. The IEDD when occurring in CIS-based strategies will result in an upward or downward frequency shift in one ear compared to the other ear as shown in Table 1 for a 22-channel vocoder. The inter-aural frequency differences resulting from the ‘n-of-m’ processing can be larger than that of IEDD because of the discrete band selection across the ears in the ‘n-of-m’ processing as plotted for the 0 mm IEDD condition in Fig. 5. Therefore, in the present study, the effect of IEDD could have been masked by the larger effects of independent band selection. Further studies could be planned to probe the effect of IEDD in ‘n-of-m’ processing with binaurally symmetric band selection in comparison to the independent band selection. Vocoder simulations are frequently used in CI research to study the specific parameters while minimizing the confounding effects that are typically present in actual CI users as listed by Kan et al. [1]. The vocoder simulations also allow the investigation of electrode parameters without the need for surgical alterations. However, vocoders are not perfect acoustic models of CI [55, 56] and the generalization of the current study findings to real-life bilateral CI listening must be done with prudence.

## Conclusions

The effect of IEDD on the use of spatial cues for vocoded speech recognition in noise was investigated using a sinewave vocoder that simulated the ‘n-of-m’ strategy in CI. The inter-aural place frequency mismatches resulting from IEDD of 1.5 mm and 3 mm were not found to influence speech recognition in noise and SRM. Irrespective of the presence or absence of IEDD, the simulated bilateral CI with ‘n-of-m’ processing reduced the speech recognition in noise and resulted in diminished SRM. Despite the advantages of the ‘n-of-m’ strategy in overcoming the channel interaction effects, the RMS amplitude-based-band selection interferes with the binaural processing. The findings of the study emphasize the need for minimizing the effects of independent band selection in sound coding strategies for optimizing the binaural advantages of BiCI. Also, the study needs to be extended to actual bilateral CI users for generalizing the findings.

## Data Availability

The datasets generated during and/or analysed during the current study are available from the corresponding author on request.
